# Spinal injury in alpine winter sports: a review

**DOI:** 10.1186/s13049-019-0645-z

**Published:** 2019-07-19

**Authors:** Sebastian Frederick Bigdon, Jan Gewiess, Sven Hoppe, Aristomenis K. Exadaktylos, Lorin M. Benneker, Paul Gilbert Fairhurst, Christoph E. Albers

**Affiliations:** 1 Department of Traumatology and Orthopaedic Surgery, Inselspital University of Bern, Freiburgstrasse, 3010 Bern, Switzerland; 2 Department of Emergency Medicine, Inselspital University of Bern, Freiburgstrasse 16C, 3010 Bern, Switzerland

**Keywords:** Trauma, Alpine injuries, Spine injuries, Review

## Abstract

**Introduction:**

Alpine winter sports have become increasingly popular over recent decades, with a similar increase in accident incidence. This review provides an overview of the most recent literature concerning spinal injury epidemiology, mechanisms, patterns and prevention strategies in the context of alpine winter sports.

**Material and methods:**

The PubMed, Cochrane Library, and EMBASE databases were searched using the keywords spine injury, alpine injury, spine fracture, skiing injuries, snowboard injuries. 64 published studies in English and German met a priori inclusion criteria and were reviewed in detail by the authors.

**Results:**

There are various mechanisms of injury in alpine winter sports (high speed falls in skiing, jumping failure in snowboarding) whilst regionality and injury severity are broadly similar. The thoracolumbar spine is the most common region for spinal injury. Spinal cord injury is relatively rare, usually accompanying distraction and rotation type fractures and is most commonly localised to the cervical spine. Disc injuries seem to occur more commonly in alpine winter sport athletes than in the general population.

**Discussion:**

Despite awareness of increasing rates and risks of spinal injuries in alpine winter sports, there has been little success in injury prevention.

## Introduction

Alpine winter sports have become increasingly popular over recent decades with ever-increasing numbers of winter resorts as well as greater accessibility. This trend is mirrored in the media as well as international competitions. Several risk factors are associated with accidents such as crowded resorts, lack of risk awareness at high speeds, and technically challenging manoeuvers. Amongst skiers and snowboarders, Corra et al. observed a rate of severe injuries of 0.2294 per million uphill rides per year [1]. The most frequent among these was traumatic brain injury, followed by spinal injuries [1]. Spinal injuries frequently occur in combination with other body regions [2, 3]. Whilst the overall injury rate seen with skiing and snowboarding has decreased, the rate of spinal injuries has plateaued or slightly increased [4, 5]. The most frequently observed spinal injuries amongst skiers and snowboarders are vertebral fractures [4]. Less than 1% of sports-related spinal cord injuries fully recover by hospital discharge [6]. Reported fatality rates in skiing and snowboarding injuries range from 0.8 to 3% [2, 7, 8].

This review provides an overview of the most recent literature concerning spinal injury epidemiology, mechanisms, patterns and prevention strategies in the context of alpine winter sports.

## Methods - skiing

**Fig. 1:**
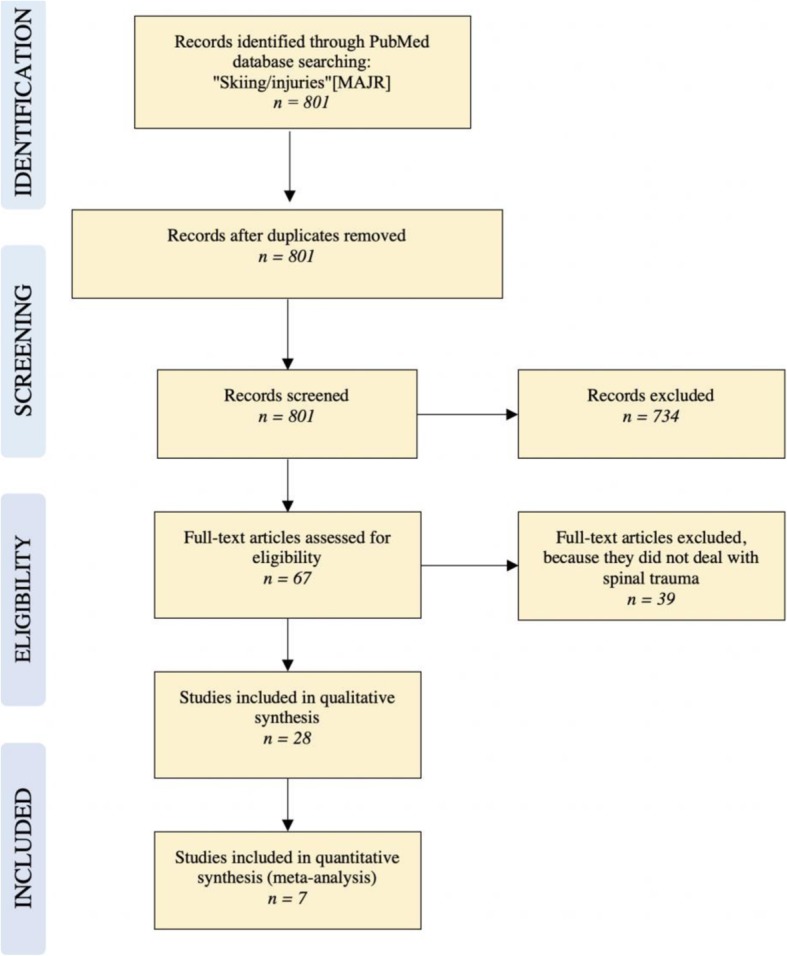
Flow diagram for study inclusion and exclusion

A literature search was performed using the PubMed database. The string “Skiing/injuries”[MAJR] yielded 801 records. These records’ titles and abstracts were screened for “disc”, “back”, “spine”, “spinal”, “freestyle”, “racers” and “racing”. From this search, 28 eligible studies were identified for qualitative analysis and seven studies provided data for a quantitative analysis. The literature search algorithm according to PRISMA Guidelines is illustrated in Fig. 1.

### Results - recreational alpine skiing

#### Epidemiology

Spinal injuries from recreational alpine skiing have been well studied, demonstrating an increasing trend in spinal injury frequency [9, 10]. Whilst the overall injury rate ranges from 1.5 to 6 per 1000 skier days [11, 12], the incidence of spinal injuries ranges from 0.001 to 0.01 per 1000 skier days [13–15]. Concurrent spinal injury amongst injured skiers ranges from 1.4–13.4% [3, 7, 16]. A similar incidence is reported in the paediatric patient population ranging from 7 to 15% [17, 18]. The mean reported age of skiers who sustained spinal injury, however, ranges from 26.7 to 41 years [3, 7, 8, 14, 16, 19]. Skiers suffering spinal injuries are predominantly male [3, 7, 8, 14, 16]. The reason for this predominance remains unclear and requires further elaboration. Of all skiing injuries, about 3% of these can be classified as severe (ISS > 16) [1]. Amongst severely injured skiers the prevalence of spinal injuries ranges from 35 to 42% [1, 7, 20]. When the spinal cord is affected, spinal injuries are found to be causal for resultant fatalities [21].

#### Mechanism of injury

The commonest mechanism of injury is falling [4, 5, 14, 16, 17]. Skiers tend to fall forwards leading to cervical spine hyperextension. This might partly explain the increased relative frequency of cervical spine injuries in skiers compared to snowboarders [22]. Falls involving axial loading most commonly lead to a burst fracture morphology [9].

Skiing accidents more frequently involved collision than snowboarding accidents [7, 17, 23], which generally are associated with more severe injury types [24].

#### Spinal injury patterns

Whilst trends have been observed, there is no absolute consensus in the literature regarding the typical site of spinal injury in skiing accidents. Retrospective studies included in this study analysed a total of 3309 spinal injuries (Table 1). The lumbar spine represents the most commonly injured site in the majority of these studies (range 30.1–64.8%) [3, 5, 7, 8, 14, 16, 19].

**Table 1:** Injury region of spinal injuries in alpine skiers

Author	Investigation Period	Number of injuries	cervical	thoracic	lumbar	sacral	coccygeal
Tarazi et al.	1994–1996	36	30.6	27.8	38.9	0	2.8
Wasden et al.	2001–2006	1137	23.2	31.1	35.1	10.6	0
Yamakawa et al.	1988–2000	91	3.3	19.8	64.8	4.4	7.7
De Roulet et al.	2007–2014	1353	26.6	33.7	30.1	9.6	
Hubbard et al.	2000–2008	482	40.7	24.1	35.2	–	–
Wick et al.	2000–2011	210	28.6	42.9	28.6	–	–

**Table 2:** Injury types in skiers

Author	Investigation period	m:f (%) mean age	Fractures included	Mechanism	Compression Type (%)	Burst Type (%)	Distraction Type (%)	Rotational Type (%)	Other Fractures (%)	percentage and site of SCI^a^
Gertzbein et al.	2005–2010	- 34y	61	-	63.9	26.2	8.2	1.6	–	–
Yamakawa et al.	1988–2000	67: 33 26.7y	91	fall	85.7	14.3	0	0	12.1 sacrococcygeal 1.1 spinous process 27.5 transv. Proc. 1.1 cervical facet 2.2 odontoid 2.2 tear drop	9.9 cervical
Tarazi et al.	1994–1996	70: 30 34.5y	36	fall	40	60	0	0	13.9 sacrococcygeal 0 spinous process 11.1 cervical facet 2.8 odontoid 2.8 tear drop	24 cervical

^a^*SCI* Spinal Cord Injury

In contrast, Hubbard et al. found the cervical spine to be the most commonly affected region [3]. Cervical spine involvement was reported in 40.7% whilst the lumbar spine accounted for only 35.2% of injuries.

Wick et al. reported fractures of the thoracic spine to be the most frequent [10]. This finding is reflected in the majority of the literature studied, with thoracic spine involvement being at least the second most common region affected (*R* 19.8–31.1%) [7, 16, 19]. Injuries with thoracic spine involvement tended to be more severe [8].

Amongst spinal injuries, vertebral body fractures predominate [14, 16]. Two studies reported these as primarily compression type fractures [16, 19] (see Table 1). In contrast, Tarazi et al., described compression type fractures in only 40% whilst burst fractures accounted for nearly 60% of the injuries studied [14]. The more severe distraction type fractures and rotation type fractures were rarely reported [19].

**Table 3:** Spinal fracture patterns in snowboarding injuries

Author	Investigation Period	Number of injuries	cervical	thoracic	lumbar	sacral	coccygeal
Tarazi et al.	1994–1996	27	18.5	22.2	37	14.8	0
Wasden et al.	2001–2006	81	17.2	29.7	42.9	9.9	0
Yamakawa et al.	1988–2000	252	2	21.8	69.4	3.2	3.6
De Roulet et al.	2007–2014	1216	23.6	33.2	34.5	8.6	
Hubbard et al.	2000–2008	113	25.3	27.1	47.6	–	–
Masuda et al.	1997–2009	19	31.6	68.4		–	–
Ishimaru et al.	2005–2012	431	1.6	22.7	61	4.6	7
Franz et al.	2000–2006	10	–	–	60	–	–

Yamakawa et al. reported transverse process fractures in over a quarter of skiing injuries [16]. By contrast, Tarazi et al. did not describe the occurrence of transverse process fractures but commented on the high incidence of cervical facet fractures [14].

Spinous process, odontoid peg and tear drop fractures are seldom reported in skiing injuries [14, 16].

In general, many studies lack systematic fracture classification. Only three studies sufficiently characterised a total of 188 spinal fractures from skiing accidents [14, 16, 19] (see Table 1).

**Table 4:** Injury types in snowboarding injuries

Author	Investigation period	m:f (%) mean age	Fractures included	Mechanism	Compression Type (%)	Burst Type (%)	Distraction Type (%)	Rotational Type (%)	Other Fractures (%)	percentage and site of SCI^a^
Ishimaru et al.	2005–2012	65.7 26.3	431	jump	82.8	14.5	2.7	–	–	
Gertzbein et al.	2005–2010	- 34	51	–	80.4	19.4	0	0	–	–
Masuda et al.	1997–2009	94.7 24.5	19^b^	jump	0	31.6	0	68.4	–	89.5 thoracolumbar
Yamakawa et al.	1988–2000	68.5 22.3	252	jump	91	9	0	0	6.8 sacrococcygeal 0.8 spinous process 39.9 transverse proc. 0.8 cervical facet 0.8 odontoid 2 tear drop	6.7 cervical
Tarazi et al.	1994–1996	100 22.4	27	jump	36.8	63.2	0	0	14.8 sacrococcygeal 7.4 spinous process 3.7 cervical facet 0 odontoid 0 tear drop	9 cervical

^a^*SCI* Spinal Cord Injury, ^b^ Only fractures with SCI were included in this study

The incidence of spinal cord injuries (SCI) amongst skiers has increased over the last two decades [9, 25]. Neurological deficits were most commonly reported in the context of cervical spine fractures [3, 10, 12, 13, 20, 26]. Hyperflexion of the cervical spine may also lead to isolated spinal cord injury without concomitant fracture or subluxation [27], especially in pediatric patients e.g. as seen in SCIWORA Syndrome (Spinal Cord Injury Without Radiographic Abnormality). Cervical SCI without skeletal injury was described by Yamakawa et al. in 2.2% of skiers who sustained spinal injuries [16].

The reported prevalence of neurological deficits with spinal cord injury amongst skiers varied greatly depending on the cohort studied, ranging from 0.93–24% [3, 9, 14, 16, 19]: Spinal fractures were associated with neurological deficits in roughly a third of the cases analysed by Reid et al. [9]. Gertzbein et al., who decribed 54 thoracolumbar vertebral fractures from skiing accidents, reported no neurological deficits of any kind [19].

Again, neurological injury and outcome were not systematically classified according to a recognised scoring system such as the ASIA-Score. As the inclusion criteria were very diverse, comparison of available data is difficult, and neurological impairment stratification in skiing injuries is not possible. Tables 1 and 2 summarize the current data for the region of injury and the pattern of injury in alpine skiing.

#### Injury prevention strategies

##### Education

Prevention should include education concerning risk factors such as fatigue and alcohol leading to poor decision-making and decreased coordination. Behaviours such as speed reduction, avoidance of technically challenging tricks, use of appropriately fitted equipment should be encouraged. Listening to music whilst downhill skiing should be avoided [28]. Slope design should incorporate injury prevention as a priority with the aim of reducing overcrowding, clear demarcation of obstacles, and creation of dedicated terrain parks with instruction of safe jumping techniques [4]. Skiing injuries in adolescents occurred with highest frequency in the afternoon which may point to fatigue as a possible risk factor for injury [17, 25]. One study suggested introducing instruction courses for optimising both ski course design as well as skiing technique with ample breaks to prevent fatigue [17].

In a meta-analysis of 12 studies, helmet use was found to significantly reduce the risk of head injuries in skiers and snowboarders without any additional increase of neck injuries [29, 30]. The benefit of spinal protection devices is controversial, with some authors arguing they reduce the incidence of back and spinal injuries [4, 17]. In one survey, 76% of winter sports participants felt that spinal protection devices conferred protection [31]. 29% of participants regularly wore a spinal protection device [13]. Stainsby et al., however, found that nearly one third of Canadian ski coaches surveyed believed that spinal protective devices conferred little or no benefit in preventing back injuries [32]. Knöringer et al. demonstrated that designs of commercially available spinal protection devices do not address the commonest biomechanical injury mechanisms (hyperflexion/hyperextension, rotational or axial compression) [13]. In addition, spinal protection devices provide no cervical spine protection and provide little support in high energy trauma situations [13]. Paradoxically, the resulting increased rigidity of the thoracic spine may potentially lead to more severe cervical spine injury.

### Results - freestyle skiing

#### Epidemiology

In a retrospective study, Brooks et al. demonstrated that back injuries occurred with higher frequency in terrain parks than on slopes [11]. Terrain park injuries occurred most likely in 13 to 24-year-old self-rated expert males who owned their equipment and wore a helmet [11].

In several international competitions freestyle skiing yielded a higher injury rate amongst the disciplines featured (Vancouver 2010 and Sochi 2014) [33, 34] and the shortest average time to injury in terms of elapsed activity time (Granada Winter Universiade 2015) [33]. Flørenes et al. reported an injury rate of 15.6 injuries per 1000 runs, of which one third were characterised as severe [34]. The highest incidence was found in halfpipe skiing, (23.9 injuries per 1000 runs) followed by aerial, ski cross and moguls [34].

#### Mechanism of injury

Whilst detailed analysis of injury mechanisms and typical spinal injury patterns in freestyle skiing has not been performed, some conclusions can be drawn: the majority of back injuries in freestyle skiing tend to occur in the lower back, followed by the upper back and the cervical spine [34]. However, in the cervical spine, injuries involved muscles, tendons and ligaments only, whereas bony injuries occurred both in the upper and the lower back [34].

#### Prevention

The epidemiologic data point to a high level of risk associated with freestyle skiing. In general, the prevention measures suggested for alpine skiing are similar to those for freestyle skiing.

### Results - Freeskiing, ski touring, snowkiting and telemarking

#### Epidemiology

Freeskiing is, by definition, predicated upon risk-taking and thrill-seeking behaviour [35]. Among slope tourers, Ruedl et al. reported an injury rate of 6 per 1000 tours. The average age of skiiers in this category was 38.8 years[28]. With telemark skiers, injuries were more likely to occur in ski resorts than in backcountry areas [36].

#### Mechanism of injury

Backcountry skiers are inherently at increased risk of injury from avalanches, crevasse falls and falls from a height. Regarding slope-tourers, falling was the most common mechanism of injury [28]. In snowkiting, speeds of over 100 km/h and jump heights of up to 10 m above ground are reported, which predisposes participants to high-energy injuries [37]. Moroder et al. (*n* = 80) reported an injury rate of 8.4 injuries per 1000 [37]. In telemark skiing, spinal injuries are commonly reported, occurring in 12.1% of all injuries [38].

#### Spinal injury patterns

The regionality of spinal injuries as well as typical injury patterns in these disciplines has not been described in the literature to date.

#### Prevention

Injuries in slope tourers occurred with higher frequency in participants who used spinal protection devices and in those who listened to music whilst touring [28]. Half of the injured snowkiters investigated by Moroder et al. used a spinal protection device [37].

### Results - professional alpine skiing

#### Epidemiology

Since spinal injury patterns of professional skiers are comparable with those of recreational skiers [39, 40], we focused on chronic back pathology in this cohort. Witwit et al. demonstrated high rates of radiological intervertebral disc pathology in young elite skiers (82% in skiers and 54% in controls) [41]. However, there was no significant difference in lifetime prevalence of back pain and MRI abnormalities in skiers showed no correlation with development of back pain [41].

#### Mechanism of injury

Radiological abnormalities of the spine are frequently reported in athletes who undergo repetitive spinal strain e.g. disc degeneration, disc herniation, apophyseal ring injury, and pars interarticularis fractures [42]. Spörri et al. described a typical spinal loading pattern with combined frontal bending, lateral bending and torsion in a loaded spine creating a high-stress environment for intervertebral discs. This spinal loading pattern is associated with the development of lower back pain [43, 44]. The combination of flexion with torsion was shown to reduce nuclear pressure required for radial tears that involve cartilaginous endplate failure in bovine models [45].

Moreover, torsional movements increased the disc wall’s resistance to radial tears that do not involve cartilaginous endplate failure [36].

Chan et al. investigated the biological response of the intervertebral disc to a physiological magnitude of torsion, as a function of the duration of applied torsion and recorded a statistical significant reduction of cell viability in nucleus pulposus cells to below 70%, when torsion was applied for 8 h per day in an artificial model using intact bovine caudal intervertebral discs [38]. Furthermore, increased time of torsion led to down-regulation of MMP-13, and significantly decreased disc volume [38]. Application of torsion-compression for 2–4 h per day tended to increase the glycosaminoglycans/hydroxyproline ratio, thus indicating that optimal load duration may be capable of promoting matrix synthesis [38].

#### Spinal injury patterns

Todd et al. compared the radiological parameters of the spino-pelvic sagittal alignment in 75 young elite skiers and 27 healthy non-athletes from plain radiographs taken in the long-standing position [39]. The study found a significant difference in the sagittal vertebral axis and a significantly higher prevalence of Type I spinal curvature according to Roussouly [39] among elite skiers, which has been associated with increased disc degeneration in the thoracolumbar region.

To date, no literature exists which characterises the distribution, frequency and severity of chronical spinal injuries in skiers.

#### Prevention

In a kinematic study, Spörri et al. investigated the effect of increased gate offset on overall trunk kinematics (including frontal bending, lateral bending, torsion angle) and their resulting ground- reaction forces and compared these between giant slalom and slalom skiers [44]. In slalom, ground-reaction force peaks were significantly lower with gate offset, whereas in giant slalom, an increase of gate offset did not result in any significant force difference. They concluded that in order to reduce the magnitude spinal loading in slalom skiers, reduced gate offsets should be avoided [44]. Interestingly, this finding runs contrary to frequent anecdotal claims by athletes. Furthermore, prevention measures in giant slalom should include reduction of the magnitude of frontal bending and lateral bending in the loaded trunk by superior core stability or the use of lumbar corsets [44]. In a similar study, Fasel et al. found standing height to be a potential measure to reduce the skier’s overall back loading, though this may have a smaller preventive effect compared to those benefits conferred by increased gate offsets [40].

## Methods - snowboarding

**Fig. 2:**
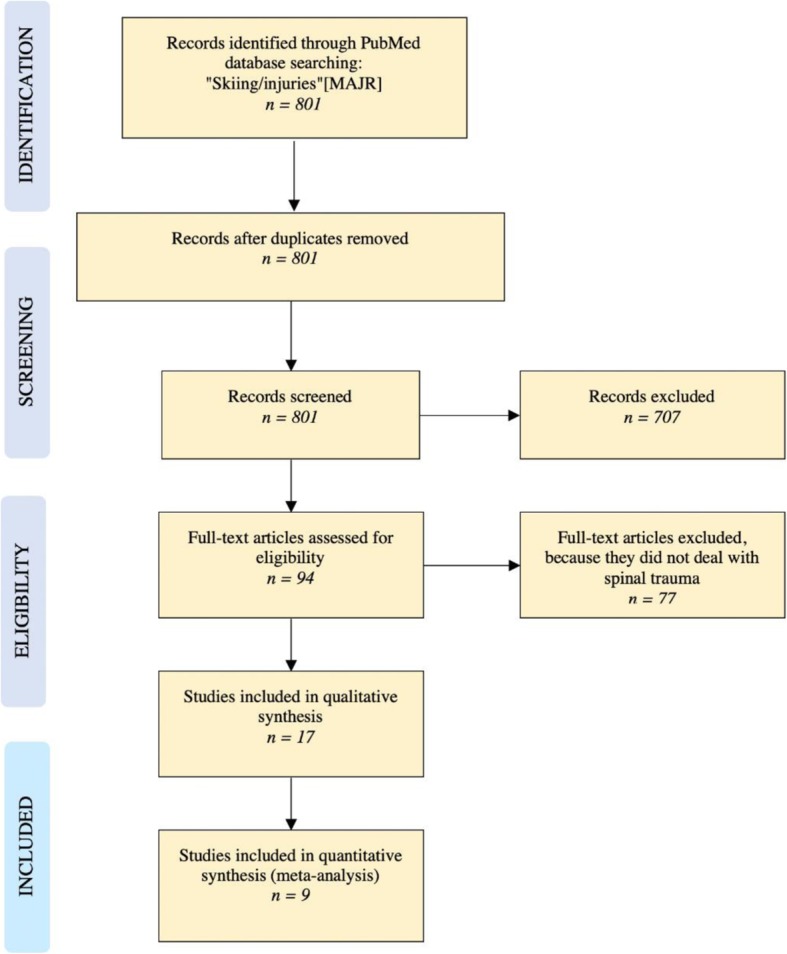
Literature search algorithm for snowboard injuries

A literature search was performed using the PubMed database. The string “Skiing/injuries”[MAJR] yielded 801 records. These records’ titles and abstracts were screened for “snowboard”, “snowboarding” or “snowboarder”. The 94 remaining records’ abstracts were screened for “disc”, “back”, “spine” or “spinal”. Seventeen eligible studies were identified for qualitative analysis and 9 studies provided data for a quantitative analysis. The literature search algorithm according to PRIMA Guidelines is illustrated in Fig. 2.

### Results - recreational snowboarding

#### Epidemiology

Because snowboarders have fixed boot-bindings and tricks involving catching air are common, typical injury characteristics are substantially different to other alpine sports. Snowboarding represents the most dangerous sport after football and ice hockey [41]. Snowboarders sustain more injuries which are typically less severe than with skiers [1, 46], although spinal injury rates are higher [4] [25]. According to a Japanese study, about 80% of snowboarders who had undergone spinal surgery returned to work but none returned to playing sports at an average follow-up of almost 4 years [47].

The prevalence of spinal injuries amongst injured snowboarders ranged from 2% to approximately 20% [3, 7, 16, 41, 45]. Regarding severely injured snowboarders with an ISS > 15, the prevalence of spinal injury of up to 47% was reported [1, 8]. High incidences of spinal injury were also reported in child and adolescent snowboarders [18].

Spinal fractures in snowboarders are frequent. Gertzbein et al. reported a likelihood for sustaining a spinal fracture of 0.009% per snowboard day [19]. Unlike in skiing, snowboarders with spinal injuries were relatively young at time of injury (20 to 34 years) [3, 7, 8, 14, 16, 19, 45, 47]. Young men were most commonly affected [3, 7, 8, 13, 16, 23, 41–45]. Novice snowboarders more commonly sustained spinal injuries than novice skiers [16, 23].

#### Mechanism of injury

Jump landing failure was the leading mechanism for spinal fractures amongst snowboarders [4, 5, 16, 23, 45, 47]. Snowboarders tend to fall backwards [48], which may result in axial loading leading to anterior compression fractures [4]. Steenstrup et al. described a typical crash sequence in order of contact with the ground: snowboard, upper extremities, buttocks/pelvis, back, trunk/chest followed by the head [48]. As in skiing, snowboarding on terrain slopes is a risk factor associated with spinal injury [11, 30, 49] [16, 23]. 

#### Spinal injury patterns

The lumbar spine was reported to be the most commonly affected region for injuries and fractures. Spinal injuries were located in the lumbar spine in 34.5 to 69.4% of the cohorts studied [3, 7, 8, 14, 16, 45, 47]. The thoracic spine was the second most commonly affected site (21.8 to 43.9% of spinal injuries) [3, 7, 8, 14, 16, 45, 47]. Most authors reported a relatively high frequency of cervical spine injuries and fractures (up to 23.6% of spinal injuries in snowboarders) [7, 8, 14, 16, 45]. Sacrococcygeal spinal injury was found in 6.8 to 17.4% of snowboarders with spinal injuries [7, 8, 14, 16, 45]. The coccygeal spine was injured with higher frequency than the sacral spine [16, 45].

Overall, vertebral fractures represented the most common spinal injuries (R 48.9 to 70.3%) [14, 16, 45]. Transverse process fractures were reported in three studies [5, 16, 45]. As with skiing, spinous process fractures, cervical facet, odontoid peg, and tear drop fractures are infrequent [14, 16, 45]. Compression type fractures occur in about 80% of snowboarders with vertebral fractures [19, 45].

By contrast, a smaller study found that burst fractures predominate [14]. Distraction type and rotation type fractures occurred seldom and were reported in only one study (14.5% distraction and 2.7% rotation fractures among vertebral fractures). Snowboarders who sustained spinal cord injury and consequently underwent spinal surgery were mostly shown to have fracture-dislocations (about 70% of cases) [47]. The distribution of vertebral fracture patterns according to the AO Spine Injury Classification is comparable to that in skiers. Ishimaru et al. reported type-A fractures in 82.8%, type-B fractures in 14.5% and type-C fractures in 2.7% of their 221 cases [45].

Hubbard et al. reported SCI in 1.07% of snowboarding injuries [3]. 25% of those were younger than 18 years. A primary diagnosis of SCI in snowboarding injuries ranged from 7 to 10% [14, 16]. Spinal cord injury occurs primarily in the context of spinal fractures. Other rarer mechanisms, such as traumatic disc rupture, have been described, however [27]. Spinal cord injury resulting from fracture-dislocation is often devastating [47]. The most commonly reported mechanism for injury leading to quadriplegia is an axial compressive force applied cranially in neck flexion. Injury is further precipitated by the absence of the protective cervical lordosis and cervical vertebral alignment [25].

As with skiing, SCI also occurred in the context of cervical spine injury in snowboarders [3, 14, 16, 20, 26]. Masuda et al., however, reported SCI to predominantly occur in the thoracolumbar junction [47]. In this cohort more than 60% of snowboarders sustained severe paralysis (Frankel grades A and B) [47]. Only two of six patients with initial Frankel grade A paralysis improved to Frankel grade D paralysis during the follow-up period [47]. Nearly 70% of these cases had persistent neurological bladder dysfunction [47]. Tables 3 and 4 summarize the current data for the region of injury and the pattern of injury in snowboarding.

#### Prevention

Similar prevention strategies apply to snowboarding as to skiing. In addition, wrist guards are advisable in preventing wrist injuries frequently arising from propping. As spinal injuries from snowboarding frequently result from jump landing failure, teaching of safe jumping technique and clear terrain demarcation restricted to expert snowboarders are recommended [25]. 6.7% of snowboarders with spinal fractures wore a spinal protection device at the time of the injury, but the evidence surrounding their use is controversial [45].

### Results - professional freestyle snowboarding

#### Epidemiology

As terrain park use is associated with a higher injury risk [11, 46, 50] and given adjudication criteria in halfpipe and big air events favour height and rotation [51], injuries in professional freestyle snowboarders are arguably more frequent, of greater severity, and of differing nature to other alpine sports. Moreover, increased impairments in activities of daily living are reported in retired snowboarders [41].

Injury incidence varies widely between different alpine sports [50, 51]: Torjussen et al. found the injury rate in big air snowboarding to be highest (2.3 per 1000 runs), followed by snowboard cross (2.1 injuries per 1000 runs) and halfpipe (1.9 per 1000 runs) [51]. Lower injury incidence was found in parallel slalom and parallel giant slalom skiing (0.3 and 0.6 per 1000 runs, respectively) [51]. Russell et al. reported a similar injury distribution [50]. The injury rate was highest for jumps and halfpipe, both with 2.56 injuries per 1000 runs [50].

Spinal injuries are common in snowboarding, accounting for 13% of all injuries [51]. Furthermore, 18% of 122 overuse injuries in the study by Torjussen et al. involved back injuries [51].

#### Mechanism of injury

Falling is the most common injury mechanism in freestyle snowboarding except for snowboard cross, where collisions predominate [51].

#### Spinal injury patterns

The regionality of spinal injuries in professional freestyle snowboarders has not yet been investigated.

Torjussen et al. showed that injury patterns in professional snowboarders differ from those of recreational snowboarders [51]. Overall, professional snowboarders sustain fewer wrist injuries but more knee and back injuries [51]. Further work is required to characterize back injuries in this population.

#### Prevention

Wearing a helmet is mandatory in snowboarding competitions. However, only 30% of 258 professional athletes interviewed by Torjussen et al. reported routine helmet use, whilst 35% reported only using helmets during competitions [51]. 62% reported using spinal protection devices [51].

Active professionals reported more frequent helmet use than retired professional snowboarders (74 and 37%, respectively) [41]. Freestyle snowboard athletes used less protection equipment than snowboarders participating in speed or snowboard cross [41].

## Results - other alpine winter sports

### Tobogganing

#### Epidemiology

The mean age for Toboggan injuries is higher than might be expected (22 to 38.1 years) [52] [53] [54]. Spinal fractures in this group tend to occur in a somewhat younger subpopulation, however. Reid et al. reported a mean age of 19 years amongst 11 tobogganers who sustained spinal fractures. Approximately a third of this group was aged under 15 at the time of injury [9].

#### Mechanism of injury

The main mechanism of injury is high-speed collision, often resulting from lapses of judgement [9, 49, 53, 55]. Ruedl et al. showed that most injuries were the result of a fall following loss of control of the toboggan [54]. Increased flexion of the vertebral column typical to tobogganing increases risk of spinal injury, particularly at the more mobile thoracolumbar junction [56]. Heim et al. reported the spine as being the fourth most common injured region amongst tobogganers [52].

#### Spinal injury patterns

Most spinal injuries are localised to the lumbar spine [9, 53]. Gröber et al. reported stable L1 fractures in 3.6% of toboggan injuries without any reported neurological impairment [53]. Reid et al. reported burst fractures in the thoracolumbar spine as occurring most frequently [9].

#### Prevention

Tobogganing should be performed in designated, obstacle-free areas that are specially prepared and children should always be supervised by adults [52]. Forward speed in a sled should be moderated [57]. Furthermore, individuals should adapt their behaviour according to track, visibility and weather conditions [54]. Although becoming more popular, protective equipment is rarely worn by tobogganers [24, 37, 53, 55]. Ruedl et al. demonstrated, however, that 13.3% of injured adult tobogganers wore spinal protection devices [54]. Alcohol is likely to be a risk factor in its effect on judgement capacity [54].

## Discussion

Alpine winter sport injuries usually involve high energy trauma and are associated with severe spinal trauma. Spinal injuries most commonly affect young men and occur more commonly in snowboarders than skiers. The mechanisms of injury in alpine winter sports differ (high speed falls in skiing, jumping failure in snowboarding) whilst regionality and severity of injuries are broadly similar. The thoracolumbar spine is the most commonly affected region. Compression type and burst fractures seem to be the most common spinal injuries. Spinal cord injury is relatively rare, usually accompanying distraction and rotation type fractures and is most commonly localised to the cervical spine. Disc injuries seem to more commonly occur in alpine winter sport athletes than in the general population. Despite awareness of increasing rates and risks of spinal injuries in alpine winter sports, there has been little success in prevention of these. Discrepancy between the requirements and efficacy of spinal protection devices has been reported, as they arguably do not prevent the most common spinal injury mechanisms. Whilst spinal injuries in skiing and snowboarding have been reported over many years, modern alpine winter sports such as freestyle or backcountry touring, freeride, telemarking, snowkiting have yet to be systematically studied with regard to their risk for sustaining spinal injury.

In our study, a narrative approach was used. This is mainly due to the relative sparsity and heterogeneity of data. As further literature becomes available with greater study numbers, a systemic review will doubtless provide valuable information where, for example, injury patterns and localisations have differed greatly in the various included studies. Another limitation is that the included studies were not all sufficiently indexed. This again is related to the sparsity of available data. The included studies also had individual limitations including publication bias, as well as greatly varying data collection and presentation (e.g. non-uniform fracture classification.)

## Conclusion

As the practice of alpine winter sports continues to change, so too do spinal injury patterns and incidence. Accordingly, further work is required to improve prevention and management.
